# Isolation and Degradation Characteristics of PBAT Film Degrading Bacteria

**DOI:** 10.3390/ijerph192417087

**Published:** 2022-12-19

**Authors:** Rehemanjiang Wufuer, Wenfeng Li, Shuzhi Wang, Jia Duo

**Affiliations:** 1 State Key Laboratory of Desert and Oasis Ecology, Xinjiang Institute of Ecology and Geography, Chinese Academy of Sciences, Urumqi 830011, China; 2 Xinjiang Key Laboratory of Environmental Pollution and Bioremediation, Xinjiang Institute of Ecology and Geography, Chinese Academy of Sciences, Urumqi 830011, China

**Keywords:** PBAT mulching film, nitrogen source, degradation rate, cultivation

## Abstract

In recent years, PBAT (polybutylene adipate-co-terephthalate) mulch has become one of the most commonly used biodegradable mulching films. In this paper, five potential strains of PBAT film degrading bacteria were screened from the soil sample using PBAT film as the sole carbon source. A highly efficient PBAT degrading strain JZ1 was isolated by comparing the degradation performance of PBAT mulching film identified as *Peribacillus frigoritolerans* S2313 by 16S rDNA sequence analysis. The capacity of the strain to degrade PBAT film was optimized by adjusting the cultivation conditions such as nitrogen source, pH, and inoculum volume. After 8 weeks of cultivation, the actual degradation rate of the strain to PBAT mulch film reached 12.45%. SEM (scanning electron microscopy) coupled with EDX (energy dispersive spectroscopy) analysis showed that microbial degradation is an oxidation process and is mainly due to the amorphous regions of the PBAT film. The biodegradation of PBAT film by *Peribacillus frigoritolerans* may provide a promising method for regulating the degradation progress of PBAT film in the farmlands.

## 1. Introduction

Since the introduction of plastic film mulching technology at the end of the 1970s, the use of plastic film and the area covered by it have continued to increase in China. In 2017, the amount of plastic film used in China exceeded 1.5 million tons and the area covered exceeded 20 million hm^2^ [1]. However, the amount of residual film in the soil has gradually been increasing due to the increase in the mulching service time and its low recovery rate. The pollution of residual plastic film in some areas has become severe with an average amount of 4~20 kg/hm^2^ [2]. Xinjiang is the province with the largest use of plastic film and covered cultivation area in China. In 2017, the total area covered by the plastic film in Xinjiang was 3.7978 million hm^2^, and the amount of plastic film used was 0.219 million tons [3], accounting for 14.6% of the total amount of plastic film used in China. Currently, most agricultural mulching films are made of polyethylene materials, whose chemical structure is stable and difficult to degrade. The mulch film left in the farmland affects soil structure and function, reduces soil fertility and the germination rate of the seeds, further affects the quality of subsequent crops, and seriously hinders the sustainable development of the agricultural industry [4,5].

In recent years, the biodegradable mulching films including PLA (polylactic acid), PCL (polycaprolactone), PBS (polybutylene succinate), PBAT, and their mixtures have been widely used in farmlands in an attempt to combat plastic film contamination [6]. PBAT mulching film has been of great interest in agriculture due to its good ductility and elongation at break, as well as good heat and impact resistance. The annual production of PBAT mulching film increased from 11,900 tons in 2011 to 33,800 tons in 2018, accounting for 7.2% of the total output of bioplastics worldwide [7,8]. The PBAT mulching film has been trialed with crops and vegetables such as cotton [9,10], corn [11,12], tomato [13,14], and others. However, the degradation of PBAT mulch in the actual application process was not ideal. For example, Wu Qiang et al. found that only small cracks appeared on the surface of PBAT mulching film in 180 days in the cotton planting process [15]. Wang et al. reported that the degradation rate of PBAT film in conventional farmland soil was only 2.3% in three months [16]. Tan et al. screened 19 kinds of bacteria from soil and found that their absolute degradation of PBAT at 30 °C was very low [17]. At present, there are a few studies on the isolation and degradation characteristics of microorganisms degrading PBAT mulching film, and the degradation rate of existing strains on PBAT was very low [18,19,20,21]. Meanwhile, the degradation of PBAT mulching film cannot be too fast because if the mulch film degrades before its induction period, its moisture and temperature-keeping functions vanish when the plant growth still needs them [22]. An induction period of about 60 days is preferable for main crops like corn and cotton in Xinjiang’s arid areas [23]. Therefore, it is necessary to find suitable efficient degradation strains to mediate the PBAT mulching film degradation process in croplands.

In this paper, a highly efficient PBAT degrading bacterium was determined by comparing five potential bacteria by the weight loss method and identified by the 16S rDNA method. The degradation conditions were optimized by adjusting the cultivation conditions such as the content of nitrogen source, the pH value, and the inoculum amount. Then, the PBAT degradation potential was assessed under the optimal degradation condition in 8 weeks. Finally, SEM-EDS analysis was applied to characterize the degraded PBAT film by this strain. 

## 2. Materials and Methods

### 2.1. Materials

#### 2.1.1. Biodegradable Film and Source of Degradation Bacteria

The type of film sample used in this test is PBAT biodegradable film, provided by Xinjiang Lan Shan Tun He New Materials Co., Ltd. (Xinjiang, China). The strain was isolated from the soil of an agricultural cotton field in Shihezi City, which had been polluted by the plastic film for a long time.

#### 2.1.2. Test Instrument

The required instruments include a vertical pressure steam sterilizer (Shanghai Shenai, LDZM-80KCS–II), thermostatic oscillator (Cristal, IS-RDV3), air drying oven (Shanghai Jinghong, DHG-9023A), biochemical incubator (Bowen, SPM-168), ultrasonic cleaner (Kunshan Shumei, KQ-250B), PCR instrument (SimpliAmp), microplate reader (Epoch), electrophoresis instrument (Powerpac Basic), and a gel imager (BioradGel Imager).

#### 2.1.3. Culture Medium

Inorganic salt medium (g/L): KH_2_PO_4_ 1.0, Na_2_HPO_4_ 1.5, NH_4_Cl 2.0, CaCl_2_ · 2H_2_O 0.1, KCl 0.2, MgSO_4_ · 7H_2_O 0.2, pH 7.2–7.4.

Screening medium (g/L): inorganic salt medium + PBAT pieces (1 cm×1 cm) 1.0.

LB medium (g/L): peptone 10, yeast powder 5, NaCl 10, pH 7.0.

Solid medium (g/L): LB medium 18, agar 20.

### 2.2. Test Method

#### 2.2.1. Soil Sampling and Film Pretreatment

The soil sample was collected from an agricultural cotton field in Shihezi city, Xinjiang region, China by using a narrow stainless shovel. Approximately 5 kg of soil specimens were collected from a depth of 15 cm in different locations and thoroughly mixed. The mixed soil sample was promptly brought to the laboratory, passed through a 60-mesh sieve, placed in a vacuum tube, and stored at 4 °C.

Film pretreatment: firstly, the biodegradable mulching film was cut into square sizes of 1 cm × 1 cm and 5 cm × 5 cm (weight 20.2 ± 0.2 mg), and soaked in 75% ethanol for 0.5 h before rinsing with deionized water 3 times. Then, the pieces were put in the digital display ultrasonic cleaner for 5 min before drying them in the air-drying oven for 24 h at 60 °C. In the end, the pieces were put under the ultraviolet sterilization lamp for 15 min for sterilization [24].

#### 2.2.2. Screening and Isolation of PBAT Film Degrading Bacteria

1.0 g of a soil sample was added to 99.0 mL of inorganic salt medium with 0.1 g of pretreated PBAT film (1 cm × 1 cm), and shaken at 28 °C, 130 rpm for 7 days. The supernatant was taken from the soil suspension in the previous step and inoculated to LB solid medium by using the dilution coating method, and incubated in the biochemical incubator for 48 h at 30 °C. Then, well-separated single colonies with different colors and shapes observed on the plates were picked, and further purified by repeat streaking [25,26]. Finally, five strains were selected through different morphological characteristics and named as JZ1, JZ2, JZ3, JZ4, and JZ5.

#### 2.2.3. PBAT Film Degradation Test

Inorganic salt medium with 1% tryptone was prepared and 3 pieces of pretreated biodegradable mulching film (5 cm × 5 cm) were added into each medium. The purified strains were inoculated into the culture medium one by one with glass rods, sealed with gauze and transferred into the thermostatic oscillator, and cultivated at 28 °C, 130 rpm for 7 days. The biodegradable mulch film was taken out after the degradation process and cleaned with the same pretreatment method mentioned above before measurement. The quality of the mulch film was accurately measured by using an electronic balance after drying. The degradation rate of the mulch film was calculated as follows (See Formula 1).

(1) $$V=\frac{V_{0}-V1}{V_{0}}\times 100\%$$

where: *V*—degradation rate of the mulch film (%);

*V*_0_—weight of biodegradable mulching film (mg);

*V*_1_—weight of biodegradable mulch film after 7 days of degradation (mg).

The strain with the highest degradation rate was finally selected for further study by comparing the degradation rates of the strains. Preparation of inoculum: the selected strain was inoculated into 100 mL of LB medium and cultured for 12 h on a shaker at 28 °C and 130 rpm. The OD_600_ (initial optical density) of the inoculum was adjusted to 1.0.

#### 2.2.4. Identification of Strain JZ1

The 16S rRNA sequence of strain JZ1 was sequenced. Common primers 27 FAGTTTGATCMTGGCTCAG and 1492 RGGTTACCTTACTACTACTACTAT were used to amplify the 16S rRNA gene. The PCR reaction system is: template (DNA 20–50 ng/μL) 0.5 μL, 10 × Buffer (with Mg^2+^) 2.5 μL, dNTP (2.5 mM) 1.0 μL, Taq Plus DNA Polymerase (5 U/Μl) 0.2 μL, 27 F/1492 R (10 μM) 0.5μL, ddH_2_O 25.0 μL. PCR reaction temperature was 95 °C for 5 min; 94 °C for 30 s, 57 °C for 30 s, 72 °C for 5 min; 72 °C for 10 min, 30 times. The amplified products were sequenced by Biotech (Shanghai) Co., Ltd., and the sequencing results were sent to the ribosome database for homology comparison. A phylogenetic tree was established by combining MEGA6 software with the adjacency method [27].

#### 2.2.5. Optimization of the Degradation Conditions

Firstly, different ratios (0%, 0.5%, 1.0%, 1.5%, 2.0%, and 2.5%) of tryptone as a nitrogen source were added to the basic salt medium, respectively. Then, the pretreated biodegradable mulch film was added and inoculated with strain JZ1. PBAT film was taken out after 7 days of shaking at 28 °C, 130 rpm. The degradation rate was calculated after cleaning and drying by the above methods. After determining the optimal amount of tryptone, the pH value of the culture medium was adjusted to 4, 5, 6, 7, 8, and 9, respectively, and the optimal pH value was determined by calculating the degradation rate of the PBAT film after 7 days cultivation. Then, the optimal inoculum amount of the strain was determined by changing the inoculation amount in the culture medium (0 mL, 0.5 mL, 1.0 mL, 1.5 mL, 2.0 mL, 2.5 mL) with the optical tryptone content and pH. Finally, the degradation experiment of PBAT film was carried out over a period of 8 weeks to analyze the degradation capacity of strain JZ1 on biodegradable mulching film under optimal conditions. 

#### 2.2.6. SEM-EDX Analysis

The PBAT film was soaked in 75% ethanol for 0.5 h, rinsed with deionized water 3 times, and put in the air-drying oven for 24 h at 60 °C before SEM-EDX analysis. A scanning electron microscope (Zeiss Super 55VP, Oberkochen, Germany) was used for analyzing the morphological changes on the surface of the PBAT film samples. Then, an energy-dispersive X-ray spectrometer (Bruker XFlash 5010, Karlsruhe, Germany) was applied to confirm the changes in the elemental composition and content of the film samples.

### 2.3. Quality Assurance and Quality Control

Stainless steel or glass materials were used in the sampling, processing, and analysis. All dishes, flasks and beakers used in the experiment were washed three times with deionized water. PBAT films were stored in closed spaces to prevent contamination from airborne contaminants. All PBAT film pieces were soaked in 75% ethanol, and washed 3 times with deionized water before and after the degradation tests. All experiments were performed in triplicate. 

### 2.4. Statistical Analysis

Data analysis was performed using Microsoft Office 2016 (Microsoft, Redmond, OR, USA) and OriginPro 9.1 (Originlab, Northampton, MA, USA). The *t*-test statistical method was applied to determine whether there were significant differences among different groups. The significance level for the analysis was set at *p* < 0.05. 

## 3. Results

### 3.1. Degradation Capacity of Potential Degrading Bacteria to PBAT

**Figure 1:**
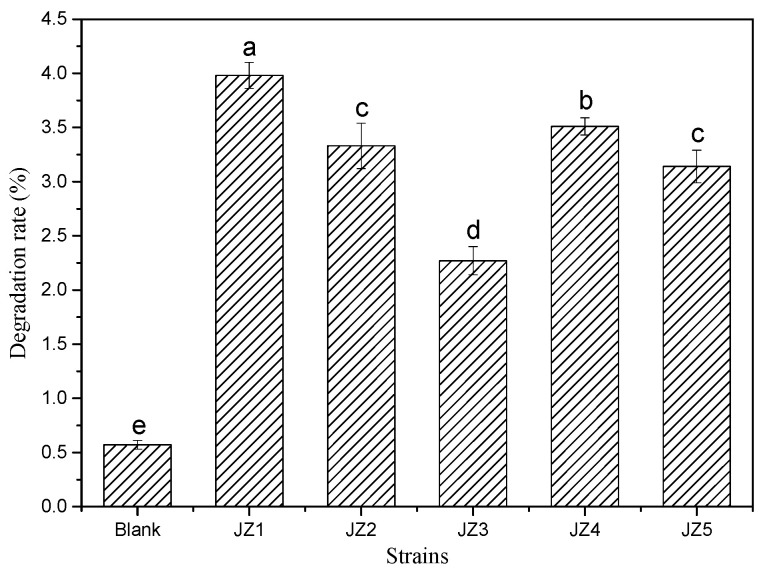
Degradation rate of PBAT film by strains in basic salt + 1% tryptone medium; a–e are symbols indicating the significance of the difference.

Figure 1 shows the degradation efficiency of PBAT film by different strains in basic salt + 1% tryptone medium after 7 days of cultivation. As shown in Figure 1, five strains (JZ1, JZ2, JZ3, JZ4, and JZ5) had different degrees of degradation efficiency on PBAT mulch. The degradation rates were 3.98%, 3.33%, 2.27%, 3.51%, and 3.14%, respectively, which are significantly higher than the degradation rate of PBAT mulch in the blank experiment (0.57%). The degradation rate is in order of JZ1 > JZ4 > JZ2 > JZ5 > JZ3. Among the five different strains, JZ1 had the highest degradation rate in the medium, which was significantly higher than the other strains (*p* < 0.05). Therefore, strain JZ1 was identified as the dominant degradation strain and selected for further study.

### 3.2. Identification of PBAT Film Degrading Strain

**Figure 2:**
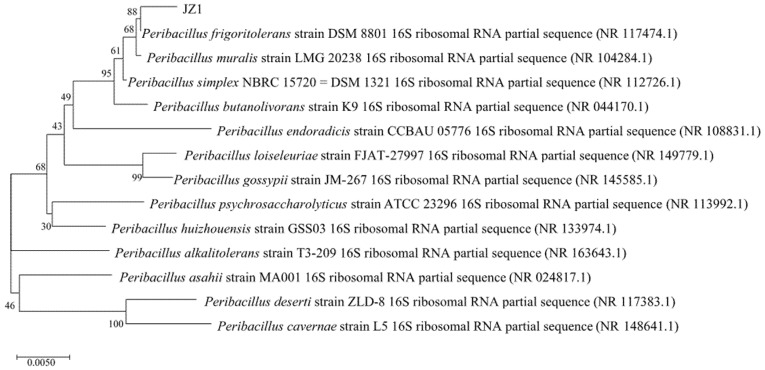
Construction of phylogenetic tree by strain JZ1 based on adjacency method.

PCR amplification was carried out with universal primers of bacteria, and the amplified products were sequenced by using the genomic DNA of strain JZ1 as the template. The obtained 16S rRNA gene sequence length was 1201 bp after splicing. The 16S rDNA sequence of strain JZ1 was registered in the ribosomal database (NR117474.1), and Blast similarity analysis was carried out with the sequence in the NCBI database. It was found that the nucleotide sequence homology of strain JZ1 and Peribacillus frigoritolerans strain LPB69 was 99.4%. The phylogenetic tree was constructed by selecting 10 16Sr RNA gene sequences with high similarity and using MEGA6.0 software and the neighbor-joining method, as shown in Figure 2.

As shown in Figure 2, strain JZ1 and Peribacillus frigoritolerans DSM8801 belong to the same branch, which indicates that they have the closest genetic relationship with Peribacillus frigoritolerans, and it can be confirmed that the strain is Peribacillus frigoritolerans S2313. 

### 3.3. Effect of Different Culture Conditions on the Degradation Rate of PBAT

#### 3.3.1. Effect of Different Tryptone Content on the Degradation Rate of PBAT Film

**Figure 3:**
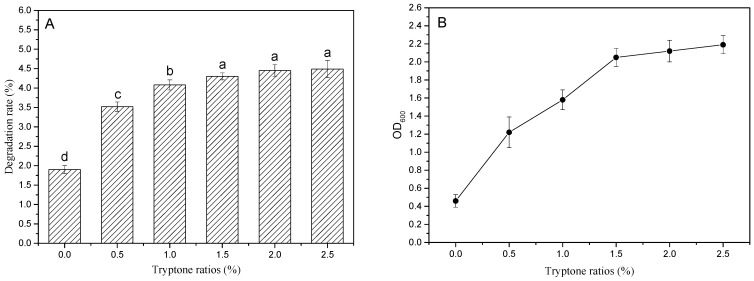
Effect of different ratios of tryptone on the degradation rate of PBAT film (**A**) and the change of biomass (OD_600_) in the medium (**B**); a–d are symbols indicating the significance of the difference.

Figure 3 shows the impact of adding different ratios of tryptone on the degradation efficiency of PBAT mulching film and the change in biomass. As shown in Figure 3A, the degradation rate of the PBAT film shows a trend of gradual increase with the increase in tryptone content in the range of 0–2.5%. The degradation rate of PBAT film was 1.90% in the basic salt medium without tryptone and significantly increased to 4.30% when the tryptone content increased to 1.5%. However, the increases in the degradation rate were not significant (*p* > 0.05) with further increases in tryptone content (2.0% and 2.5%). This trend is basically consistent with the growth trend of strain JZ1 under the conditions of different tryptone content (see Figure 3B). The biomass in the medium significantly increased from 0.46 to 2.05 (*p* < 0.05) when the tryptone content increased from 0% to 1.5%. The biomass increases were not significant with the further increases in the tryptone content to 2.0% and 2.5%, indicating that the degradation process of biodegradable mulch film by strain JZ1 is a growth-associated process.

#### 3.3.2. Effect of Different pH on Degradation Rate of PBAT Film

**Figure 4:**
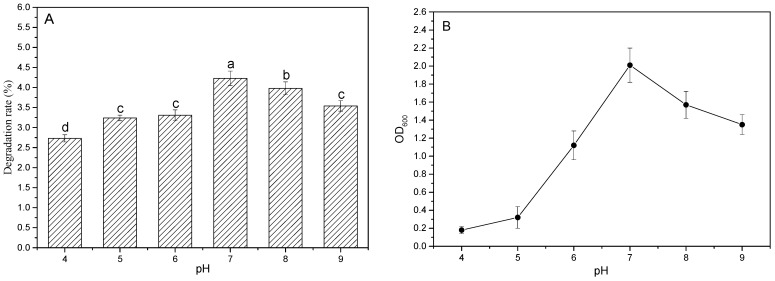
Effect of pH values on the degradation rate of PBAT film (**A**) and change of biomass (OD_600_) in the medium (**B**); a–d are symbols indicating the significance of the difference.

Figure 4 shows the impact of different pH values on the PBAT film degradation rate and the change of biomass in the basic salt + 1.5% tryptone medium. As shown in Figure 4A, the degradation rate of the biodegradable plastic film shows a trend of increasing first and then decreasing with the increase of pH value. The degradation rate of the PBAT film was only 2.73% when the pH value was 4 and reached its peak of 4.21% when the pH became neutral (7.0), which was significantly higher than the values in other acid–alkaline culture conditions (*p* < 0.05). This changing of the trend of the film degradation rate is consistent with the OD_600_ changes in different pH values (Figure 4B). OD_600_ was only 0.18 when the pH was 4, and increased dramatically to 2.01 when the culture medium became neutral, then slowly decreased to 1.35 when the pH further increased to 9.0. The similar changing trend between the biomass and the degradation rate of the PBAT film indicates that the strain grows best under neutral conditions and is directly related to the degradation of PBAT film. 

#### 3.3.3. Effect of Different Inoculum Amounts on the Degradation Rate of PBAT Film

**Figure 5:**
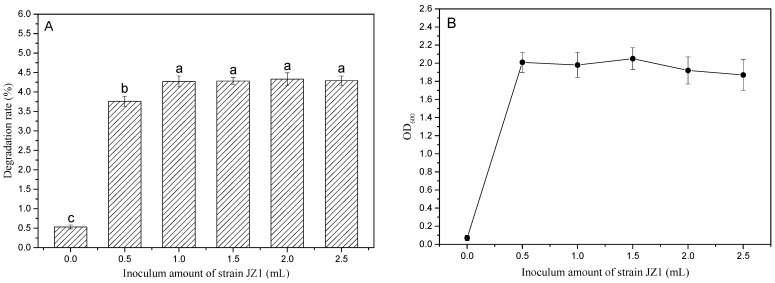
Effect of different inoculation amounts on degradation rate of PBAT film (**A**) and the change of biomass (OD_600_) in the medium (**B**); a–c are symbols indicating the significance of the difference.

Figure 5 shows the impact of different inoculum amounts on the PBAT film degradation rate and the change of biomass in the basic salt + 1.5% tryptone medium. As shown in Figure 5A, there was only 0.53% of PBAT film loss in the blank medium (without inoculation of the strain JZ1). The degradation rates significantly increased to 3.76% and 4.27% with the addition of 0.5 mL and 1.0 mL inoculum, and stabilized at this level with further increases in the inoculum amount (1.5–2.5 mL). Figure 5B shows that OD_600_ was only 0.07 in the blank medium and increased sharply to 2.01 (with 0.5 mL inoculum) and kept at this level until the inoculation amount increased to 1.5 mL before slightly decreasing to 1.92 and 1.87 (*p* > 0.05) when the inoculum amount further increased to 2.0 mL and 2.5 mL. Therefore, the inoculum amount of 1.0 mL was chosen in this study.

#### 3.3.4. Effect of Strain Culture Time on PBAT Film Degradation Rate under Optimized Conditions

**Figure 6:**
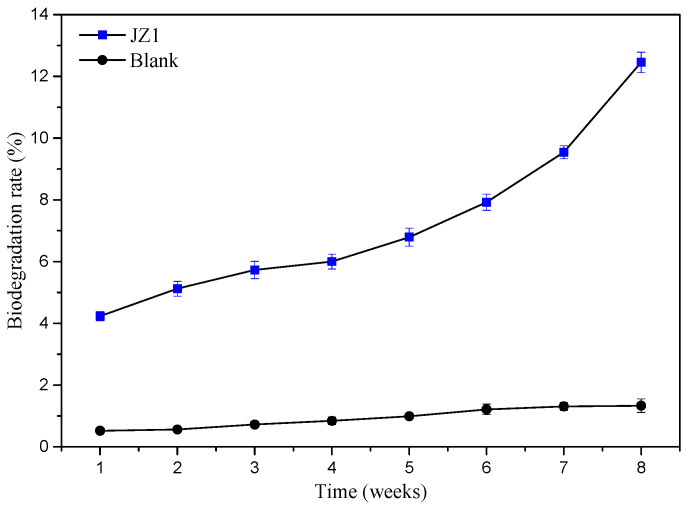
The trend of PBAT film degradation rate with and without inoculation of JZ1 over a period of 8 weeks.

Figure 6 shows the trend of PBAT mulching film degradation rate over 8 weeks under the optimized conditions. As shown in Figure 6, the degradation rate of PBAT mulching film shows a trend of gradual increase over time. In the first week, the degradation rate of PBAT film degraded by JZ1 was 4.23% while only 0.52% of film loss occurred in the blank medium. The degradation rate of the plastic film increased relatively slower from the second to fifth week with a total increase of 2.56%. From the sixth week, the degradation rate of the PBAT film increased significantly and reached 12.45% by the end of the eighth week, which indicates that the PBAT film entered its induction period at this stage. Only 1.33% of PBAT film was degraded over the whole period of 8 weeks in the blank medium without the inoculation of JZ1. 

#### 3.3.5. SEM-EDS Analysis

**Figure 7:**
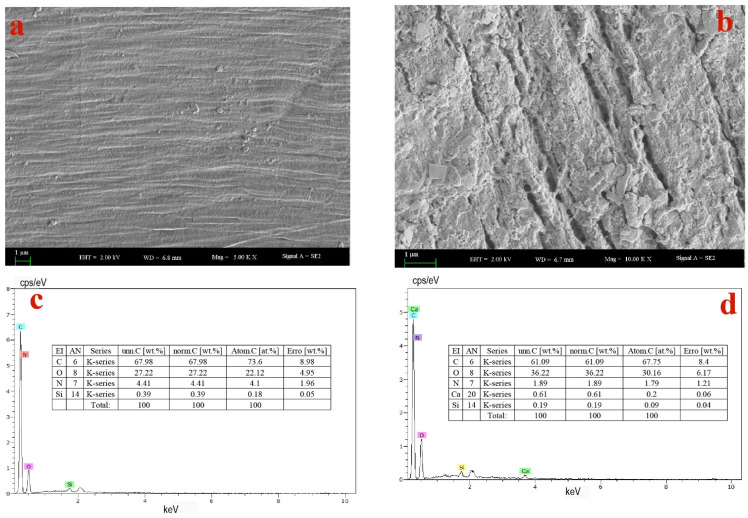
SEM images and EDX spectra of the PBAT film before and after the 8 weeks degradation by strain *Peribacillus frigoritolerans* S2313; (**a**) SEM image before the degradation; (**b)** SEM image after the 8 weeks degradation; (**c**) EDX spectra before the degradation; (**d**) EDX spectra after the degradation. Typical images and spectra are shown from one of the triplicate examinations.

As shown in Figure 7a, the original PBAT film surface is smooth and flat. After 8 weeks of degradation, the PBAT film surface was damaged and irregular cracks and defects appeared on the surface, and the roughness on the surface significantly increased (Figure 7b), indicating that the molecular structure of the PBAT film sample has been damaged and obvious degradation has occurred. It can also be observed from Figure 7b that the surface degradation of the PBAT mulching film sample was uneven during the degradation process, which may be caused by the different degradation rates of amorphous and crystalline regions of PBAT [28]. 

Figure 7c,d shows the changes in element composition and content before and after PBAT degradation. It can be seen from Figure 7c,d that the content of C element in the PBAT film decreased from the original 67.98% to 61.09%, while the content of the oxygen element increased from the original 27.22% to 36.22% after degradation. The C/O ratio decreased from 2.50 to 1.69 after degradation, indicating that the degradation process of PBAT is also an oxidation process.

## 4. Discussion

The PBAT-degrading bacterium we isolated from the soil sample in our research was first defined as *Brevibacterium frigoritolerans* in 1953 [29] and reclassified as *Peribacillus frigoritolerans* later [30]. This bacterium has been reported to effectively degrade organophosphorus pesticide phorate [31,32,33,34] in soil and paracetamol [35] in the liquid mineral salt medium. This is the first study that reports the degradation of the PBAT mulch film by *Peribacillus frigoritolerans*. Most plastic-degrading bacteria can degrade plastics by secreting degrading enzymes such as urease, laccase, peroxidase, and lipase. The enzyme genes can use oxygen molecules to introduce unstable oxygen-containing functional groups into the stable chemical structure of plastics [36,37]. The introduction of new polar functional groups into PBAT plastic film structure is also conducive to the adhesion of microorganisms on the film surface and the decomposition of the film, thus further accelerating the degradation role of the strains [38,39]. Jin Mengmeng et al. isolated *Brevibacterium frigoritolerans* GD44 from the radioactive soil in the Xinjiang region and identified the genes encoding enzymes related to the degradation of organophosphorus compounds such as esterase, phosphotransferase, C-P lyase, and alkaline phosphatase by sequence analysis [40], which indicates that these enzymes might also be responsible for the fracture of ester bonds in the PBAT film structure 

In our research, the degradation rate by this bacterium on PBAT film at an ambient temperature reached 4.30% in 7 days in neutral conditions and increased to 12.45% after 8 weeks of cultivation. So far, a few microorganisms have been reported to be capable of degrading PBAT or PBAT mixed materials. For instance, Kasuya et al. found that the degradation rates of PBAT film by bacteria NKCM2511 and NKCM2512 isolated from soil were 1.4% and 1.2%, respectively [19]. Muroi et al. separated three strains of bacteria from soil that can degrade PBAT film. Among them, strain NKCM3201 has the highest degradation capability (1.2% in 10 days) [20]. Huo Xiangdong et al. isolated strain XJSL2 (*Sphingopyxis ginsengiso*) from the soil samples of Shule County in Xinjiang. The degradation rate of PBAT was 0.92% after 60 days of degradation [27]. Liu Jiaxi et al. screened six strains of PBAT plastic-degrading bacteria from the soil samples. After 8 weeks of degradation, the PBAT film degradation rates by both strains RD1-3 and N1-2 exceeded 6% [21]. The high temperature was demonstrated to boost the degradation of PBAT film by bacteria. For example, Zhang Min et al. isolated PLX/PBAT degrading strain XJ11 (*Delftia tsuruhatensis*) from a cotton field soil sample and the degradation rate reached 6.87% at 37 °C in 7 days [25]. Under the composting condition of 58 °C, the degradation rate of the PBAT film reached 64.3% in 48 days [41]. Hao Jia et al. reported that Stenotrophomonas sp. had a 10.14% of PBAT film degradation rate by adding 2% of 1,4- butanediol in an SM medium at 37 °C in 5 days [42]. In comparison with bacterial degradation, yeast or fungi have been reported to have much higher, even complete, degradation of PBAT films. For instance, Fungi *Paraphoma Chrysanthemicola* B47-9 had a 99.2% of PBAT film degradation rate in 7 days [43], while the yeast strain, *Cryptococcus sp*. MTCC 5455 achieved complete PBAT film degradation at 25 °C within 9 days [44]. To sum up, the degradation rate of the PBAT film mainly depends on factors such as the type of the microorganism, the culture conditions, and the longevity of the cultivation period, etc. 

In order to further study the degradation characteristics of the PBAT film, we used SEM-EDS to analyze morphological and elemental changes before and after 8 weeks of degradation by *Peribacillus frigoritolerans* S2313. SEM images showed that the PBAT film surface was damaged and irregular cracks and defects appeared on the surface. EDX analysis showed that the content of oxygen elements significantly increased after 8 weeks biodegradation. The increase in oxygen atom content led to the decrease in hydrophobicity and the increase in roughness and fracture on the PBAT surface [21,45]. 

The induction period of PBAT mulch film is very important for the growth of crops and vegetables, and the necessary longevity depends on the growing crop types. If the induction period is too short, the mulch film breaks too early and its moisture and temperature-keeping functions vanish when the plant growth still needs them. If the induction period is too long, the mulch film degrades too slowly and the film residues will affect plantation and soil function [22]. An induction period of about 60 days is preferable for main crops like corn and cotton in Xinjiang’s arid areas. The branches and leaves of the crops grow big enough to keep moisture in the soil environment after two months of growth [46]. Our results showed that the PBAT film degradation significantly accelerated in eight weeks, which indicated that the PBAT film entered its induction period at this stage. Therefore, this bacterial strain might be very suitable for mediating the PBAT degradation process in croplands. 

## 5. Conclusions

Our results demonstrated that the bacterial strain *Peribacillus frigoritolerans* S2313 isolated from a cotton field could effectively degrade PBAT film. Under optimized conditions of nitrogen source, pH, and inoculum amount, the degradation rate by this strain on PBAT film reached 12.45% in 8 weeks. The substantial increase in the PBAT film degradation in the eighth week indicated that the PBAT film entered its induction period, which is suitable for main crops like cotton and maize in Xinjiang’s arid areas. SEM-EDX analysis showed that microbial degradation is an oxidation process and is mainly due to the amorphous regions of the PBAT film. However, in field conditions, the degradation process of the mulch film is affected by many factors including temperature, radiation, moisture, soil and crop types, and film composition. Therefore, the isolated strain *Peribacillus frigoritolerans* S2313 should be demonstrated in larger-scale field experiments considering the above-mentioned environmental factors and used to mediate the PBAT film degradation process in the field.

## Data Availability

The study did not report any data.
